# Understanding Mycotoxin Contamination Across the Food Chain in Brazil: Challenges and Opportunities

**DOI:** 10.3390/toxins11070411

**Published:** 2019-07-15

**Authors:** Marta H. Taniwaki, John I. Pitt, Marina V. Copetti, Aldir A. Teixeira, Beatriz T. Iamanaka

**Affiliations:** 1 Food Technology Institute, ITAL, C.P. 139, Campinas - SP, CEP 13078-170, Brazil; 2 CSIRO Agriculture and Food, P.O. Box 52, North Ryde, NSW 1670, Australia; 3 Departamento de Tecnologia e Ciência de Alimentos, Centro de Ciências Rurais (CEP), Universidade Federal de Santa Maria (UFSM), Santa Maria RS 97105-900, Brazil; 4 Experimental Agrícola do Brasil Ltda, São Paulo - SP, CEP 04105-001, Brazil

**Keywords:** coffee, cocoa, Brazil nuts, mycotoxins, ochratoxin A, aflatoxins, fungi, trade barrier

## Abstract

Brazil is one of the largest food producers and exporters in the world. In the late 20th century, the European Union program for the harmonization of regulations for contaminants in food, including mycotoxins, led to the examination of mycotoxin contamination in foods at a global level. The problem of the rejection of food by the European Union and other countries became a Brazilian national priority because of economic and food safety aspects. Ochratoxin A in coffee and cocoa and aflatoxins in Brazil nuts are examples of the impact of technical trade barriers on Brazilian foods. To overcome these threats, several strategies were undertaken by Brazilian and international organizations. In this context, the Codex Commission on Food Contaminants (CCCF) has emerged as a forum to discuss with more transparency issues related to mycotoxins, focusing on establishing maximum levels and codes of practices for some commodities and mycotoxins to ensure fair trade and food safety. Our experience in investigating and understanding mycotoxin contamination across the food chains in Brazil has contributed nationally and internationally to providing some answers to these issues.

## 1. Introduction

**Figure 1:**
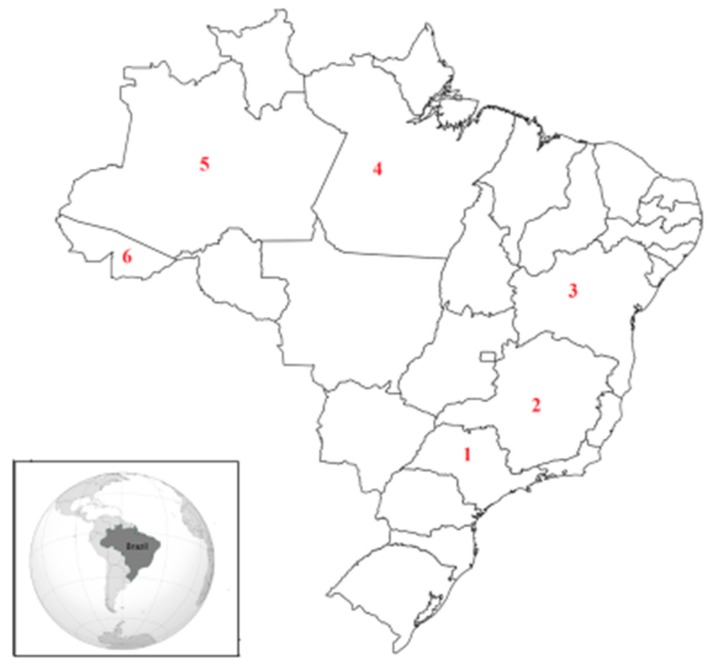
Map of Brazil indicating the localization of the main states’ producers of coffee: Sao Paulo (1) and Minas Gerais (2); cocoa: Bahia (3) and Para (4); and Brazil nuts: Amazonas (5) and Acre (6).

Brazil is the fifth largest country in population (around 200 million people) and geographical area, and the largest in terms of arable land with 851 million hectares. It hosts five major climatic types: Equatorial, tropical, subtropical, temperate and semiarid and is one of the world’s biggest food producers and exporters (Figure 1). It is the largest producer and exporter of coffee, sugar and orange juice, and is highly ranked in the production and export of soybeans, maize, ethanol, pork, beef and poultry among others [1]. Brazilian agribusiness represents 23% of Brazil’s gross domestic product (GDP), one third of all employment and almost 40% of exports [2]. Only a fraction of its land is exploited but the country produces a highly diverse array of agricultural goods, leading the country to a unique position in the global agricultural sector in the medium to long term. Brazil has an abundant supply of natural resources including water, large areas of cheap arable land with the potential to double crop areas, varied soils, and a favourable climate that allows for two or more harvests per year [1].

In spite of this potential, major obstacles exist from both internal and external factors. Of the internal factors, significant needs exist to improve productivity and infrastructure. Of the external, the international markets have a strong impact on the Brazilian economy, as the export of commodities is often very restricted by international trade policies based on food safety. In the early 1990s, the European Union program for the harmonization of regulations for contaminants in food, including mycotoxins, brought about the necessity to examine mycotoxin contamination in foods at a global level. The rejection of food by the European Union and other countries became a Brazilian national priority because of economic and food safety aspects.

In 2007 the Codex Committee on Food Additives and Contaminants in Foods (CCFAC) was divided and the new Codex Committee in Food Contaminants (CCCF) was established as a forum to discuss with more transparency issues related to mycotoxins focusing on establishing the maximum levels for some mycotoxins (Table 1) and the code of practices for several commodities and mycotoxins [3]. Before 2011 Brazil only had limits for aflatoxins in peanuts, maize, dry and fluid milk. The establishment of maximum levels for some mycotoxins in the European Union and the CCCF forum showed the necessity of revising the Brazilian regulation for mycotoxins, as some foods rejected by international markets resulted in an entrance of contaminated foods in Brazilian markets. This fact raised the urgency of establishing limits for mycotoxins in several foods consumed in Brazil. In 2011 the Brazilian Surveillance Agency (ANVISA) established regulations for six mycotoxins, aflatoxins, ochratoxin A, fumonisins, zearalenone, deoxynivalenol and patulin, in more than 20 food categories and the previous regulations dealing with human food were revoked in Brazil [4].

Mycotoxins, especially ochratoxin A in coffee and cocoa and aflatoxins in Brazil nuts, are examples of the impact of technical trade barriers on Brazilian goods. To overcome these threats, several strategies were undertaken by Brazilian and international organizations that will be discussed below.

## 2. Coffee

### 2.1. Coffee as a Global Commodity

Coffee is a primary product in world trade; the second largest traded commodity in value only after oil. It plays a vital role in the balance of trade between developed and developing countries. Coffee is grown and exported by more than 50 developing countries, the major consumers being USA, Europe, Brazil and Japan. Coffee cultivation, processing, trading, transport and marketing employ over 100 million people worldwide. Coffee is a part of Brazilian history. Brazil is the largest green coffee bean producer and exporter in the world [5], accounting for 35% of the supply of coffee. Coffee is the fifth most exported item in Brazilian agribusiness, responsible for almost 5% of export revenues from agricultural products. The Brazilian Ministry of Agriculture, Livestock and Supply (MAPA) projections suggest that Brazil will continue to be the largest producer and exporter of coffee for the next decade [6].

### 2.2. Emergence of Ochratoxin A (OTA) in Coffee

The occurrence of ochratoxin A (OTA) in raw coffee was first reported by Levi et al. [7]. Initial data suggested that high level of destruction of mycotoxins would result during the roasting process. However, occurrence of OTA in market samples of roasted coffee, as well as in coffee beverages, was reported after improvements in detection methods [8,9,10]. As a consequence, the European Commission’s Scientific Committee on Food considered that OTA in coffee could be a contaminant of concern due to its potential of genotoxicity and carcinogenicity, and it would be prudent to reduce exposure to OTA as much as possible [11]. Pressure from European authorities prompted coffee importers, and coffee-producing countries to survey OTA in coffee products, and to investigate the fate of OTA during the production, handling and manufacture of raw coffee. Surveys were conducted on the presence of OTA in Europe on raw coffee of different origins and on roasted and soluble coffee produced and sold in Europe [9,12,13,14]. Later surveys in several other countries have confirmed the presence of OTA in raw, roasted and soluble coffee [15,16,17,18]. Extensive sampling of raw coffee from all origins has shown that OTA contamination may be more frequent in some areas, but that no producing country is entirely free from contamination [19,20].

In 1997 the Brazilian Consortium for Coffee Research and Development (CBPD Café) was established to improve the competitiveness of the Brazilian coffee agribusiness. This consortium of government sectors, academia and industry enabled intensive research of the whole coffee chain to diagnose the true situation on OTA contamination in Brazilian coffee.

The work that was carried out by our group aimed to investigate: (i) The fungi responsible for OTA production; (ii) which stages of coffee production were susceptible to infection by fungi capable of OTA production; (iii) the influence of climatic conditions and processing practices on OTA production; (iv) which factors during coffee transport contributed to fungal growth and OTA production; (v) what were the effects of the roasting process on OTA destruction; and (vi) a risk assessment of OTA in coffee consumption.

### 2.3. Fungi Producing OTA in Coffee

The work carried out by us and other groups concluded that three *Aspergillus* species, *A. ochraceus, A. carbonarius* and *A niger*, are responsible for the production of OTA in coffee. While *A. niger* is a more common fungus than *A. ochraceus* or *A. carbonarius*, only 3% to 10% of *A. niger* isolates are capable of producing OTA, so this species is probably a relatively unimportant source of OTA in coffee [17,21]. *A. ochraceus* has been isolated from coffee samples around the world and was accepted as the major cause of OTA in coffee beans [17,22,23,24,25]. However, molecular studies by our group showed that two distinct species related to *A. ochraceus* were found in Brazilian coffee [26]. At the same time, with the finding that the classical concept of *A. ochraceus* consisted of three species, it was recognized that the main species producing OTA in coffee was *A. westerdijkiae* rather than *A. ochraceus* [27]. The third species in this group *A. steynii*, has also been found to produce OTA in coffee, but it is not as common as *A. westerdijkiae* or *A. ochraceus* [18,21]. For simplicity, *A. ochraceus* is used below to refer to all three species.

### 2.4. Stages of Coffee Production Susceptible to Infection by Fungi Capable of OTA Production

Evidence has shown that OTA is formed in raw coffee beans only after harvest [17]. Consequently, post-harvest handling using appropriate techniques for drying, grading, hulling and sorting to reduce defects provides the basis for producing coffee with low levels of OTA. Moreover, the management at the pre-harvest stage can minimize the amount of toxigenic spores and reduce the risks of their growth in post-harvest [28].

### 2.5. Brazilian Study Cases on OTA in Coffee

**Figure 2:**
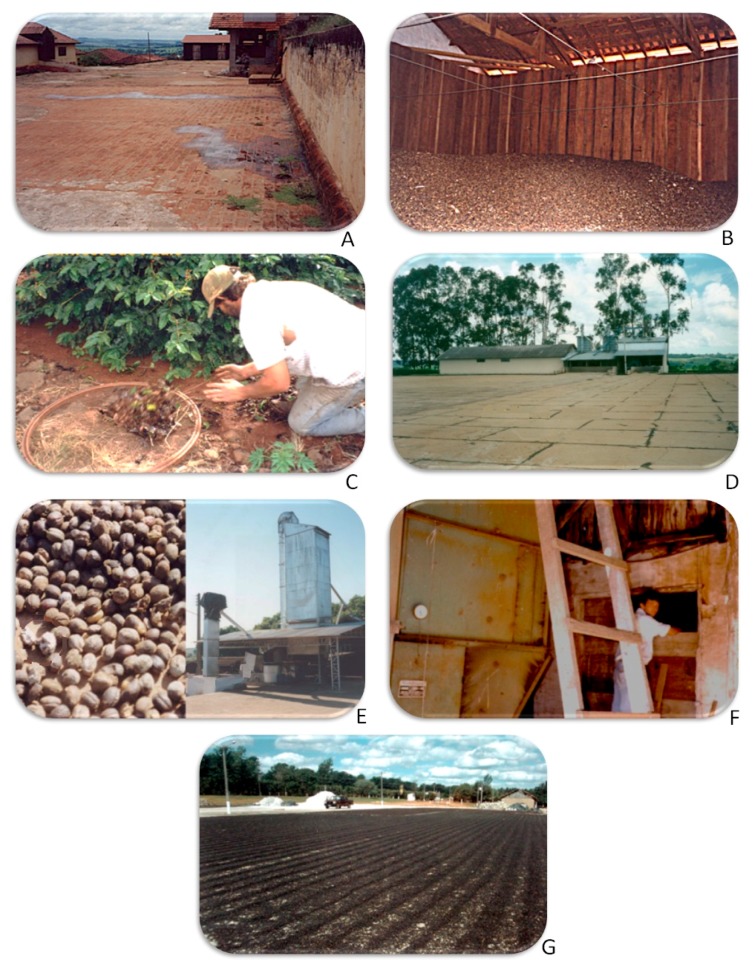
Study cases of coffee farms located in: Southwest São Paulo State, drying yard (**A**), storage (**B**), coffee collection from the ground (**C**); Northeast São Paulo State, drying yard (**D**); Western São Paulo State, drying yard and elevator (**E**), coffee dryer and storage (**F**); Western Minas Gerais State, drying yard (**G**).

Our investigations of the relationship between the formation of OTA in coffee, local climatic conditions and processing factors showed that OTA occurrence was sporadic and occurred only after harvest [17]. To illustrate these studies, six case studies where OTA was found at levels of 5 µg/kg or higher in coffee samples from farms are shown in Figure 2. Each case is summarised below:

**Case 1.** Southwest São Paulo state is a relatively cold, rainy region (average of 18 °C and 65 mm/month rainfall) of low altitude (<800 m) and generally producing low quality coffee. One sample of coffee segregated as “floaters” during processing from storage was contaminated with 110 μg/kg OTA. Investigation showed several critical points: The farm was located in a basin frequently affected by a heavy mist (Figure 2a); the coffee was turned over only infrequently during drying; in many cases the coffee layer was very thick; the barn showed signs of moisture infiltration from the ceiling and floor; and birds had access to the storage barn (Figure 2b), favouring growth of *A. ochraceus* and production of OTA. *A. ochraceus* was isolated from 30% of beans from this sample.

**Case 2.** On a second farm also in southwest São Paulo State, the agricultural practice was very poor, as coffee beans were routinely collected from the soil by raking (Figure 2c). A sample contained 6 µg/kg OTA. The source was probably *A. ochraceus*, although percentage infection was low (4%).

**Case 3.** Northeast São Paulo State is a temperate, moderate rainfall region (average of 20 °C and 30 mm/month rainfall) of relatively high altitude (800–1000 m), producing generally good quality coffee (Figure 2d). However, two samples from one farm contained excessive OTA; one from storage contained 10 μg/kg OTA, while the second, from the drying yard, contained 48 µg/kg OTA. Investigation showed that the drying yard was very small for the amount of dried coffee produced, so that, before drying, the coffee was often heaped up in the yard, waiting to be spread out. Mist was also a problem because the farm is located in a basin. The time required to dry was therefore often excessive, allowing time for fungal growth and OTA production.

**Case 4.** Western São Paulo State is a hot, rainy region (average of 21 °C during the growing and harvest season and 47 mm/month rainfall) of relatively low altitude (<800 m) and an average coffee quality (Figure 2e). One coffee sample contained 5 μg/kg OTA and was visibly moldy. *A. carbonarius* was isolated with growth perhaps favoured by the high temperature. Investigation showed that the reason for poor quality samples was a broken elevator which permitted dried coffee at the bottom to become wet.

**Case 5.** On a second farm in western São Paulo State, one sample from storage contained 10 μg/kg OTA, although OTA-producing fungi were not found (Figure 2f). The storage barn was very close to the coffee dryer, which was found to be injecting hot, humid air into the barn. The temperature of the coffee at the top of the stack was high, which caused a warm humid condition for fungal growth and OTA production.

**Case 6.** The western part of Minas Gerais State is a temperate, dry region (average of 19 °C and 15 mm/month rainfall) of high altitude (>1100 m), producing good quality coffee (Figure 2g). Samples from a farm showed neither OTA-producing fungi nor OTA contamination. This farm showed good practices at all stages.

### 2.6. Storage and Transport

Some European countries have rejected coffee beans from Brazil due to the presence of OTA. To understand which factors during coffee transportation contributed to fungal growth and OTA production, a maritime voyage from Brazil to Italy was undertaken to monitor coffee containers located in different parts of a ship [29]. On maritime routes between regions of tropical climate such as Brazil and those of temperate climate such as Italy, a common destination for coffee, temperature oscillations during transport can cause moisture transfer inside non-hermetic systems such as containers used for coffee transportation. Some factors are out of the control of both producers and exporters. Several recommendations were made by this study in order to reduce OTA production during coffee transportation [29].

### 2.7. Effect of Roasting on OTA

Studies have shown that coffee roasting removes 8 to 98% of OTA [9,30], depending on the roasting process. The low level of OTA contamination found in roasted and soluble coffee [19,20] indicates that coffee is not a major source of OTA in a diet with estimated intakes being well within safety guidelines. More research is needed on the effects of the roasting, grinding and beverage preparation on the stability of OTA. Modified mycotoxins can be formed during processing [31] but their toxicity remains unclear.

## 3. The Food and Agriculture Organization (FAO)/Common Fund for Commodities (CFC) Project

After some studies showed the occurrence of OTA in market samples of roasted coffee [8,9], a workshop was organized by the International Life Sciences Institute (ILSI) in Aix-en-Provence, France, in 1996 to discuss the significance of OTA in coffee. Together with the EU program for harmonizing regulation on mycotoxins, this workshop resulted in a project, funded by the European coffee industry and the Common Fund for Commodities, to clarify the significance of OTA in coffee and the impacts of its presence on human health and economic aspects.

The project was carried out by the Food and Agriculture Organization of the United Nations (FAO) on “Enhancement of coffee quality by the prevention of mould formation.” It included seven producing countries for implementation: Brazil, Colombia, Côte d’Ivore, India, Indonesia, Kenya and Uganda. The objective was to improve the quality of coffee through the development and sustainable implementation of a strategy that included, inter alia, training and extension programs in coffee-producing countries and also covered storage and transport practices. The project started with a small scale project in Uganda before its implementation in other countries.

### 3.1. A Mission to Uganda

With results obtained in Brazil on OTA-producing fungi and the critical points of OTA production throughout the coffee chain and presented at the 18th International Scientific Colloquium on Coffee in Helsinki [32], the opportunity to help other countries such as Uganda was considered in the light of the Brazilian experience. A mission to Uganda was undertaken in 2000 to assess points in the Uganda production chain that would favour OTA production in coffee and to train local specialists. A summary of this mission is reported below.

Coffee is the most economically important commodity in Uganda, accounting for 65% of the country’s foreign exchange earnings. It employs approximately 3 million people, the majority of whom are small-scale farmers. Usually, harvested coffee is dried in the sun on bare ground, and it may take seven to fifteen days to reduce moisture to 13%. Most farmers leave the coffee in the drying area at night. Some farmers store the coffee in a dry area during rain, but others leave it exposed, while some cover the coffee with tarpaulins in the drying area.

The coffee marketing system appears to operate as follows: Large producers (farms around or above 200 ha) own their own shelling machines and sell directly to exporters. Small producers sell their product to either small traders or shellers, who dry the coffee. These businesses then sell Fair Average Quality (FAQ) coffee to exporters. The commercialization chain from small producer to large exporter has a varying number of intermediaries, making coffee quality and profitability difficult to predict.

Several recommendations were made from this mission. Coffee quality in Uganda could be improved and fungal contamination reduced with the implementation of good practices from harvesting to storage and transport. A guide to good harvesting, storage, handling and processing published by the Uganda Coffee Development Authority (UCDA) should be easily understood by small producers and traders. The establishment of a chain of educators, identifying motivated personnel in the farming communities, is recommended. Such people could act as multipliers of good agricultural practices in their communities. Adaptation of technical information to make it more accessible to small operators, using more explanatory drawings and figures and less text would also be helpful.

During this brief visit it was found that the coffee district in Mbale was more organized and had an advantage over other districts. The likely reason was probably the existence of a cooperative, an organization that can reduce production costs and stimulate the implementation of good agricultural practices.

### 3.2. Global Strategies to Reduce OTA in Coffee

In 2006, the FAO published the final report on the project “Enhancement of coffee quality through prevention of mould formation” [33]. This report assisted the operators throughout the coffee chain to apply good agricultural practice, good practices in transport and storage and good manufacturing practices to reduce OTA formation. The Codex Alimentarius Commission on Food Contaminants (CCCF) provided a Code of Practice for the Prevention and Reduction of OTA in Coffee with guidelines to mitigate this hazard in coffee beans [34].

In 2006 the European Union [35] established the maximum level of 5 µg/kg of OTA for roasted coffee beans and ground roasted coffee and 10 µg/kg for soluble coffee (instant coffee). For green coffee, no limit was established, although several coffee companies in Europe have their own specifications for OTA levels in coffee for exporting countries.

In Brazil, work from several researchers has been showcased in seminars, workshops and informal meetings, such as field days, to transfer the information on good practice in transport and storage. This has involved public authorities, coffee producers, cooperatives, industry and academia. Several campaigns offering prizes for the best quality coffee in different producing states have contributed to improve coffee quality.

## 4. Cocoa

Cocoa, *Theobroma cacao* L., is a perenial tree originally from the Amazon rainforests of South and Central America. Today it is planted in about 50 countries in the tropics [36]. Cocoa trees are well adapted to tropical climates with high humidity and warm temperatures. West Africa concentrates most cocoa production, although significant quantities also come from Asia, and Central and South America.

**Figure 3:**
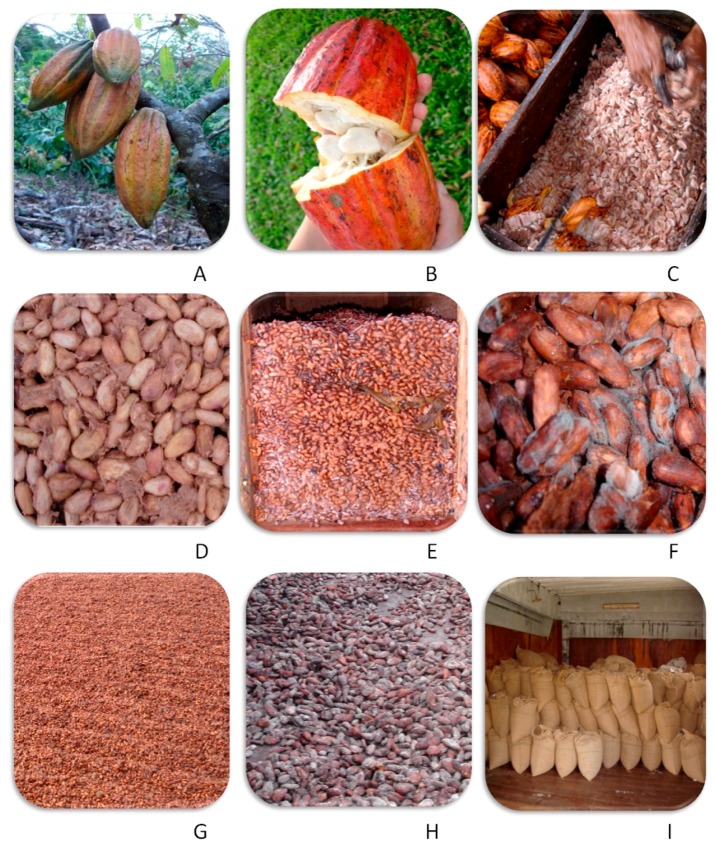
Cocoa processing steps: Pods on the tree (**A**); opening (**B**,**C**); fermentation (**D**–**F**); drying (**G**,**H**); storage (**I**).

Cocoa trees usually reach 3 to 5 m in height. Cocoa beans are produced in large pods (Figure 3a) that are harvested and opened manually with the help of knives (Figure 3b,c). After, the beans with a pulp rich in carbohydrate are piled in heaps or in wooden boxes to undergo a spontaneous fermentation for about seven days (Figure 3d–f). Microorganisms that contaminate the cocoa beans originate from workers’ hands and tools, surface of pods, banana tree leaves (cut and mixed with the beans), insects or residual mucilage present in collection baskets and equipment [37]. Yeasts and lactic or acetic acid bacteria are desirable, since they have an important role in the production of chocolate aroma precursors. These molecules are achieved after the diffusion of enzymes, alcohol and lactic and acetic acids, produced by microorganisms through the cocoa cotyledon, leading to the embryo death [38,39,40]. After the fermentation process, beans are moved to sun-drying platforms (Figure 3g,h) to reduce their moisture content, stopping microbial growth and enzyme production at the same time that some organic acids produced during fermentation are volatilized, reducing the cocoa beans’ acidity. The dried beans are commonly stored in bags (Figure 3i) until marketed. Once having arrived in processing plants, the cocoa beans are roasted, shelled and milled; then can follow two distinct processing lines producing powdered cocoa or chocolate [37].

Cocoa trees are cultivated in eight states in Brazil, with Bahia and Para (Figure 1) representing 94% of plantations in national production [41]. In the late 1970s, Brazil was the second largest producer in the world; however, production declined drastically in the 1990s, when “witches’ broom” disease spread across most cocoa plantations, reducing the production by 60%. As a consequence, Brazilian cocoa production fell to sixth in the world, so most of Brazilian cocoa production now is absorbed by domestic markets [42]. Several efforts have been made in the last few years by cocoa producers to increase their production.

### 4.1. Ochratoxin A in Cocoa and Byproducts

The first report on OTA in cocoa beans was published by Van Walbeek [43]. However, an effective analytical methodology was only developed in 1983 [44] and the first major survey for the presence of this toxin in cocoa and cocoa products only occurred in 2004 [45].

While Brazil is not a major cocoa exporter, several multinational cocoa-processing companies are established in the country, with their own standards for quality and safety, including mycotoxins. Therefore, in 2005 a project on “Cocoa mycobiota: fungi and mycotoxins from cocoa tree to chocolate” was commissioned. Because of our experience with OTA in coffee, we were asked to carry out this project with a grant from the São Paulo State Research Funding Agency (FAPESP). Objectives were: (i) To study the presence of ochratoxigenic fungi and OTA throughout the cocoa chain; (ii) to assess the effect of environmental conditions and processing practices on OTA production; (iii) to study the influence of processing on OTA reduction.

### 4.2. Ochratoxigenic Fungi and OTA in Cocoa

At harvest, beans and pulp inside a healthy pod are microbiologically sterile (Figure 3a). After the pod is opened (Figure 3b,c), it becomes contaminated with microorganisms that will contribute to the subsequent natural fermentation process. During the last days of fermentation, filamentous fungi, including the ochratoxigenic species *A. carbonarius* and *A. niger*, were found [46]. Experimental work demonstrated the importance of organic acids, especially acetic acid, produced by fermentative bacteria, in suppressing the growth of ochratoxigenic fungi and highlighted the importance of an adequate fermentation step [47]. It was also shown that OTA production increases during drying of partially depulped beans. Therefore, it was recommended that a traditional fermentation of four to seven days should be adopted, turning the mass frequently (Figure 3d) as fermentation beyond seven days can lead to fungal proliferation, mainly in the outer pulp layer [37] (Figure 3e,f).

Sun drying (Figure 3g) usually takes around seven days under good conditions, but in adverse weather can take two to four weeks. Prolonged drying increases the chance of fungi growth and spoilage (Figure 3h). *A. carbonarius* and *A. niger* were found and about 50% of samples were positive for OTA [46]. However, the levels were generally low, with a mean of 0.13 µg/kg OTA. A strong correlation was observed between the occurrence of OTA in the cocoa beans and the presence of *A. carbonarius*, indicating that this species is the major source of OTA in cocoa.

After cocoa beans reach about 6–7% of moisture content, they are stored in bags at the farm until marketed (Figure 3i). Good storage conditions are crucial to maintain the quality of the beans. It is recommended that the storage of cocoa in tropical countries should not exceed two to three months [37,48].

### 4.3. Reduction of OTA During Cocoa Processing

The process of roasting cocoa beans decreased OTA concentrations by 17% under conditions evaluated by our group [49]. It was also demonstrated that most OTA is concentrated in the shell fraction and just a small part of the toxin contaminates the nibs. Therefore, the winnowing, for segregating the shell from the nibs, is a critical point to reduce OTA contamination, responsible for about 90% of OTA reduction. In the pressing step, OTA remained bound to the cocoa cake and, consequently, the OTA concentration in the cocoa butter was very low. Moreover, mycotoxin levels in alkalized cocoa powder were lower than in untreated fractions [49,50].

The co-occurrence of aflatoxins and ochratoxin A in chocolate sold in markets revealed these two mycotoxins in at least 80% of samples, although at low levels [50].

### 4.4. Exposure and Regulation of OTA in Cocoa and Cocoa Products

Assessment of dietary intake of OTA by the European Union people concluded that cocoa contributes to around 5% of OTA intake in diet [51]. A similar dietary exposure estimation in Hong Kong concluded that chocolate contributed to 6% of the total dietary exposure to OTA [52].

In 2011, the Codex Committee on Contaminants in Food (CCCF) revised the “Discussion paper on OTA in cocoa” [52]. Information was gathered from several countries on the occurrence of OTA in cocoa and byproducts to verify the degree of contamination, the contribution of these products on human exposure to OTA through the diet, the main factors influencing OTA formation in cocoa and its fate during processing.

In 2013 the “Code of practice for the prevention and reduction of OTA contamination in cocoa” [53] was established with several recommendations for the cocoa production chain.

So far, few countries have set regulatory limits for OTA in cocoa beans and cocoa products. Tafuri et al. [54] recommended setting a limit for OTA in raw cocoa and byproducts (maximum of 1 µg/kg in chocolate, chocolate powder and drinking chocolate and 2 µg/kg in cocoa beans, cocoa nibs, cocoa mass, cocoa cake and cocoa powder) [54]. However, the European Commission has stated that it did not appear necessary at that moment [55]. Considering the high consumption of cocoa products and chocolate by children and the results published by our group [46,56], the Brazilian Sanitary Surveillance Agency (ANVISA) set limits of 10 µg/kg for cocoa beans and 5 µg/kg for cocoa products and chocolate sold in Brazil, for both OTA and total aflatoxins [4].

## 5. Brazil Nuts: *Bertholletia excelsa*

The Amazon rainforest has multiple ecosystems with huge biodiversity. The equatorial climate is hot and humid, with an average temperature of 26 °C and relative humidity of 80–95%. Brazil nuts are one of the most important products extracted from this region. Production is totally organic and environmentally sound, as no chemical products are used to control pests and weeds or for fertilizing. This ecosystem favours a unique biodiversity of fungal species different from those found in cultivated areas.

Trees of *Bertholletia excelsa* grow wild, reaching up to 60 meters, take 12 years to bear fruit and may live up to 500 years. The trees are found in groves of 50–100 individuals and the groves are separated by up to 1 km. Pollination is by wild, large-bodied bees, especially *Euglossa*, *Xylocopa* and *Bombus* species [57,58]. Fruits are spherical capsules of ligneous mesocarp and are extremely hard. Seed numbers average 18, and they have a ligneous, rough shell. The seeds (nuts) are rich in proteins, lipids and vitamins and are a source of selenium.

Brazil nut gathering has a socio-economic and environmental importance in the Amazon region, as it generates employment for the local population, contributes to forest maintenance and is the main product for export from the Amazon region.

### 5.1. The Emergence of Aflatoxins in Brazil Nuts

In 2003, the European Union restricted Brazil nut importation due to reports of elevated levels of aflatoxins [59], causing a 10% return of contaminated lots due to excessive aflatoxin contamination (unpublished data). This restriction was of great concern both economically and with regard to food safety and consumer health in Brazil.

The occurrence of aflatoxins in Brazil nuts has become a critical constraint for commercialization, as they have been reported in samples sold in several countries, including the United States [60], Japan [61], Sweden [62,63] and Brazil [64,65,66,67].

### 5.2. Fungi Producing Aflatoxins

In an investigation of the sources of aflatoxins in Brazil nuts, Calderari et al. [64] found five potential producing species. *A. flavus* was found in 29% of nuts, but unexpectedly *A. nomius* was equally common (30% of nuts). All isolates of *A. nomius* produced aflatoxins B_1_, B_2_, G_1_ and G_2_ and 46% of *A. flavus* isolates produced aflatoxins B_1_ and B_2_.

**Figure 4:**
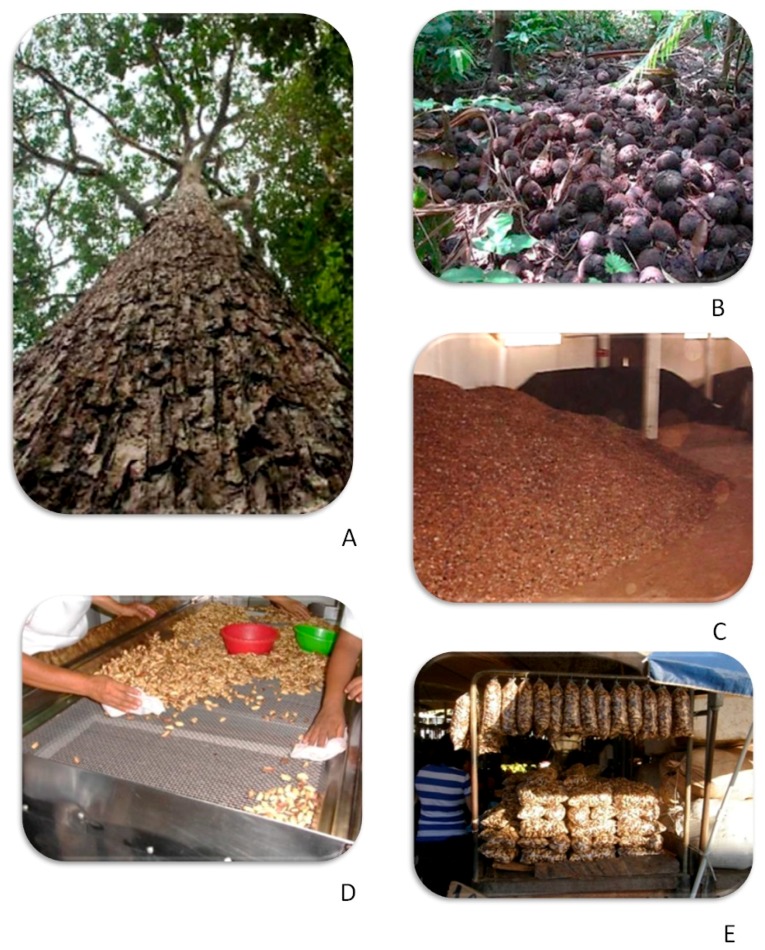
Brazil nuts value chain: *Bertholletia excelsa* tree (**A**); pods on the ground in Amazon rainforest (**B**); storage in processing area (**C**); sorting (**D**); street markets (**E**).

Brazil nuts are an extractive product, so common agricultural practices do not apply. Pods fall from trees during January to April, when the environment is very humid due to constant rain (Figure 4a,b). The pods stay in contact with soil for long periods in the forest because collectors will not work while pods are still falling due to the real possibility of serious injury. Water can readily enter the pods on the soil through the operculum, permitting fungal growth. The infection of Brazil nuts by *A. nomius* in the rainforest can reach 96% with toxin concentration reaching 20 µg/kg. Aflatoxins can be produced at any time while the pods are on the ground [66].

Collected nuts are transported to processing plants where they are sorted, dried and shelled (Figure 4c). The shelled nuts are manually sorted by size and quality (Figure 4d). The initial stage of manual/visual sorting is important to remove moldy and stained seeds and the step precedes classification by size. At the end of the drying and cooling processes, the nuts are vacuum packaged [64].

Aflatoxigenic fungi and aflatoxins have been found throughout the Brazil nut chain [66]. According to Johnsson et al. [68], growth and aflatoxin production increases rapidly between 40 and 90 days after collection of nuts before arrival at the processing plant for final drying.

### 5.3. Strategies to Reduce Aflatoxin Contamination

The low level of technology characteristic of the Brazil nut production chain and the inadequate conditions of raw material management favour the formation of aflatoxin.

To overcome these problems a series of actions were implemented by producing countries (Bolivia, Peru and Brazil) and their respective governments, research institutes, universities and non-governmental organizations to reduce aflatoxin contamination to meet international standards and to strengthen export markets.

“Safenut”, a project coordinated by the European Union was completed in 2008. Its objective was to validate an efficient and sustainable system of safety and transfer to the Brazil nut production chain. This work strengthened the capacity of local producers to meet international standards, restoring access to export markets, protected human health and reduced deforestation in the Amazon. The project recommended studies on the microbiota of Brazil nuts to better understand the infection process and subsequent aflatoxin contamination and validation of aflatoxin assay.

Our team carried out a project supported by the Brazilian Ministry of Agriculture, Livestock and Supply (MAPA) for elucidating some aspects such as the mycobiota of Brazil nuts and methods for contamination control.

As described above, it was found that control of aflatoxin at the primary stage was very difficult due to conditions found in the Amazonian rainforest. However, in processing plants, sorting was shown to be a very effective way to decrease aflatoxin levels in Brazil nuts, with reductions of more than 90% achieved. The temperature (60 °C) used for drying is not enough to destroy aflatoxins. Marklinder et al. [62] reported that consumers have the ability to visually discriminate nuts with a high content of aflatoxin from sound nuts, thus protecting themselves from consuming high levels of aflatoxins.

The highest level of aflatoxins we found in Brazil nuts was from samples sold in street markets in the Amazon (Figure 4e), with one unshelled sample containing more than 150 µg/kg and one shelled sample 140 µg/kg. [66]. These samples were ready to be eaten and posed a risk to consumers. Often the nuts sold in such markets are of low quality and price and may have been rejected by processors.

In 2010, the CCCF elaborated a code of practice for the prevention and reduction of aflatoxin in tree nuts with additional measures for Brazil nuts [69]. This code of practice recommended that Brazil nuts should be dried to a safe moisture level (<0.7 a_w_) within 10 days after collection, either in the extractivist communities or after transport to a processor. In practice this may not always be possible because of climate and logistic conditions. However, while an effective sorting system is possible to produce a safe product in processing plants, a better surveillance system is needed for Brazil nuts sold in street markets.

In 2010, the Codex Alimentarius Commission [70] recommended a maximum level for total aflatoxins in Brazil nuts for further processing of 15 µg/kg and for ready to eat of 10 µg/kg. At the same time, the European Commission [55] adopted the maximum levels of aflatoxin B_1_ and total aflatoxins in Brazil nuts for further processing of 8.0 and 15.0 µg/kg and for ready to eat of 5.0 and 10.0 µg/kg, respectively [55]. For Brazil nut-producing countries, these limits are feasible, taking into account the reality of Amazonian rainforest conditions.

## 6. Conclusions

CCCF emerged as a forum to discuss with more transparency issues related to mycotoxins focusing on establishing maximum levels for some mycotoxins and the code of practice for several commodities and mycotoxins. Our experience in investigating the origin of mycotoxins throughout the food supply chain has contributed nationally and internationally to understanding the issues raised in this paper. While OTA in coffee and cocoa and aflatoxins in Brazil nuts have not been totally eliminated, our work has contributed to radical improvements of the whole supply chain. It is a challenge but also an opportunity to improve the quality and safety of our products for domestic and international markets.

## Figures and Tables

**Table 1:** Maximum levels of mycotoxins recommended by the Codex Alimentarius Commission [3].

Mycotoxins	Commodity/Product	Maximum Level (µg/kg)	Adoption Year
Total aflatoxins (B_1_, B_2_, G_1_, G_2_)	Peanuts for further processing	15	1999
	Almonds for further processing	15	2008
	Almonds “ready to eat”	10	
	Hazelnuts for further processing	15	
	Hazelnuts “ready to eat”	10	
	Pistachios for further processing	15	
	Pistachios “ready to eat”	10	
	Brazil nuts for further processing	15	2010
	Brazil nuts “ready to eat”	10	
	Dried figs	10	2012
Aflatoxin M_1_	Milk	0.5	2001
Patulin	Apple juice	50	2003
Ochratoxin A	Raw wheat	5	2008
	Barley	5	
	Rye	5	
Deoxynivalenol	Cereal-based foods for infants and young children (dry matter basis)	200	2015
	Flour, meal, semolina and flakes derived from wheat, maize or barley	1000	
	Cereal grains (wheat, maize and barley) destined for further processing	2000	
Fumonisins B_1_, B_2_	Raw maize	4000	2014
	Maize flour, maize meal	2000	
